# Supplementary material for: The regulation of oocyte maturation and ovulation in the closest sister group of vertebrates
Source: eLife. 2019 Oct 1;8:e49062. doi: 10.7554/eLife.49062 (PMC6786877; doi:10.7554/eLife.49062)
Supplement: Figure 2—figure supplement 2—source data 1. — Relative expression values of the genes to CiUbac1 (RNA-seq and qRT-PCR data) are shown. [file elife-49062-fig2-figsupp2-data1.docx]

**Supplementary file 2.**

**Relative expression values of the genes to *Ci-ubac1* (RNA-seq data).** Related to **Figure 2-figure supplement 2.**

| **Independent experiment** | **early Stage I** | **late I-**  **early II** | **early II-**  **late II** | **late II-**  **late III** | **Stage IV** |
| --- | --- | --- | --- | --- | --- |
| ***Ci-gnrh-r1*** |  |  |  |  |  |
| 1 | 0.95 | 0.16 | 0.26 | 0.73 | 0.44 |
| 2 | 1.05 | 0.22 | 0.03 | 0.32 | 0.68 |
| ***Cio-r2*** |  |  |  |  |  |
| 1 | 0.79 | 1.69 | 1.18 | 2.87 | 5.65 |
| 2 | 1.21 | 1.15 | 0.74 | 1.93 | 7.05 |
| ***Ci-tk-r*** |  |  |  |  |  |
| 1 | 1.01 | 2.43 | 1.79 | 1.77 | 1.64 |
| 2 | 0.99 | 2.08 | 1.68 | 1.59 | 1.95 |
| ***Ci-vp-r*** |  |  |  |  |  |
| 1 | 0 | 1.49 | 2.34 | 3.17 | 1.81 |
| 2 | 0 | 0.51 | 2.61 | 3.62 | 0.98 |
| ***Ci-erk1/2*** |  |  |  |  |  |
| 1 | 0.64 | 2.08 | 3.16 | 7.12 | 7.80 |
| 2 | 1.36 | 3.53 | 3.38 | 7.51 | 8.09 |

**Relative expression values of the genes to *Ci-ubac1* (qRT-PCR data).** Related to **Figure 2-figure supplement 2.**

| **Independent experiment** | **early Stage I** | **late I-**  **early II** | **early II-**  **late II** | **late II-**  **late III** | **Stage IV** |
| --- | --- | --- | --- | --- | --- |
| ***Ci-vp-r*** |  |  |  |  |  |
| 1 | 1.38 | 2.01 | 3.15 | 6.09 | 5.34 |
| 2 | 0.66 | 1.58 | 2.41 | 5.76 | 2.77 |
| 3 | 0.96 | 0.26 | 1.63 | 1.77 | 2.68 |
| ***Ci-erk1/2*** |  |  |  |  |  |
| 1 | 0.98 | 2.05 | 2.24 | 2.55 | 3.32 |
| 2 | 1.67 | 2.04 | 2.95 | 4.64 | 4.82 |
| 3 | 0.35 | 0.91 | 1.29 | 1.24 | 2.35 |
