# Supplementary material for: The regulation of oocyte maturation and ovulation in the closest sister group of vertebrates
Source: eLife. 2019 Oct 1;8:e49062. doi: 10.7554/eLife.49062 (PMC6786877; doi:10.7554/eLife.49062)
Supplement: Figure 3—source data 1. — Percentages of GVBD and ovulated follicles after incubating with CiVP (Figure 3C) or a MEK inhibitor, U0126 (Figure 3F). Percentage of immature follicle or developed embryo incubated after in vitro fertilization using CiVP-induced follicles (Figure 3—figure supplement 1). [file elife-49062-fig3-data1.docx]

**Supplementary file 3.**

**Percentages of GVBD and ovulated follicles after incubating with Ci-VP.** Related to **Figure 3C.**

| **Independent experiment** | **Control** | **5 μM Ci-VP** | **5 μM Ci-VP (Linear)** |
| --- | --- | --- | --- |
| **GVBD rate (%)** |  |  |  |
| 1 | 50.0 | 76.9 | 57.7 |
| 2 | 50.0 | 62.5 | 60.9 |
| 3 | 36.0 | 64.0 | 36.0 |
| 4 | 32.0 | 54.2 | 41.7 |
| 5 | 22.7 | 57.9 | 54.5 |
| 6 | 46.2 | 82.6 | 57.1 |
| **Ovulation rate (%)** |  |  |  |
| 1 | 21.4 | 65.4 | 0 |
| 2 | 35.0 | 65.0 | 25.0 |
| 3 | 30.8 | 59.3 | 15.4 |
| 4 | 13.6 | 63.2 | 31.8 |
| 5 | 16.0 | 92.0 | 48.0 |
| 6 | 32.0 | 87.5 | 41.7 |

**Percentages of GVBD and ovulated follicles after incubating with a MEK inhibitor, U0126.** Related to **Figure 3F.**

| **Independent experiment** | **Control** | **10 μM U0126** |
| --- | --- | --- |
| **GVBD rate (%)** |  |  |
| 1 | 94.7 | 7.1 |
| 2 | 82.8 | 13.2 |
| 3 | 85.7 | 13.3 |
| 4 | 90.9 | 40.9 |
| **Ovulation rate (%)** |  |  |
| 1 | 47.1 | 0 |
| 2 | 65.9 | 19.0 |
| 3 | 41.9 | 6.5 |
| 4 | 62.5 | 0 |

**Percentage of immature follicle or developed embryo incubated after *in vitro* fertilization using Ci-VP-induced follicles.** Related to **Figure 3-figure supplement 1.**

| **Independent experiment** | **Immature follicle** | |  | **Pre-hatch embryo** | |
| --- | --- | --- | --- | --- | --- |
|  | **Control** | **5 μM Ci-VP** |  | **Control** | **5 μM Ci-VP** |
| 1 | 61.1 | 10.5 |  | 0 | 52.6 |
| 2 | 61.1 | 0 |  | 16.7 | 50.0 |
| 3 | 60.0 | 37.5 |  | 20.0 | 41.7 |
| 4 | 54.2 | 25.0 |  | 16.7 | 50.0 |
