# Supplementary material for: The regulation of oocyte maturation and ovulation in the closest sister group of vertebrates
Source: eLife. 2019 Oct 1;8:e49062. doi: 10.7554/eLife.49062 (PMC6786877; doi:10.7554/eLife.49062)
Supplement: Figure 4—source data 1. — Percentages of GVBD follicles after incubating with CiVP and/or a Cdc2 inhibitor, Ro-3306 (Figure 4B and C). Relative expression values of the CiCcnb and CiCdk1 genes to CiUbac1 (RNA-seq data, Figure 4—figure supplement 3A) and relative values of in vitro CiCdk1 activity are also shown (Figure 4—figure supplement 3C). [file elife-49062-fig4-data1.docx]

**Supplementary file 5.**

**Percentages of GVBD follicles after incubating with a Cdc2 inhibitor, Ro-3306.** Related to **Figure 4B.**

| **Independent experiment** | **Control** | **1 μM Ro-3306** |
| --- | --- | --- |
| **GVBD rate (%)** |  |  |
| 1 | 92.9 | 14.3 |
| 2 | 100.0 | 14.3 |
| 3 | 54.5 | 0 |
| 4 | 63.6 | 0 |
| 5 | 95.0 | 0 |
| 6 | 100.0 | 4.8 |

**Percentages of GVBD follicles after incubating with Ci-VP and Ro-3306.** Related to **Figure 4C.**

| **Independent experiment** | **Control** | **5 μM Ci-VP** | **5 μM Ci-VP**  **+1 μM Ro-3306** |
| --- | --- | --- | --- |
| **GVBD rate (%)** |  |  |  |
| 1 | 38.9 | 89.5 | 23.5 |
| 2 | 25.0 | 50.0 | 0 |
| 3 | 40.0 | 100.0 | 10.0 |

**Relative expression values of the genes to *Ci-ubac1* (RNA-seq data).** Related to **Figure 4-figure supplement 3A.**

| **Independent experiment** | **Control** | **10 μM U0126** |
| --- | --- | --- |
| ***Ci-ccn-b*** |  |  |
| 1 | 1.17 | 0.97 |
| 2 | 1.03 | 0.44 |
| 3 | 0.80 | 0.29 |
| ***Ci-cdk1*** |  |  |
| 1 | 1.30 | 1.48 |
| 2 | 0.95 | 0.39 |
| 3 | 0.75 | 0.71 |

**Relative values of *in vitro* Ci-Cdc2 activity.** Related to **Figure 4-figure supplement 3C.**

| **Independent experiment** | **Blank** | **Control** | **1 μM Ro-3306** |
| --- | --- | --- | --- |
| 1 | 1.02 | 14.45 | 7.35 |
| 2 | 1.11 | 14.48 | 7.25 |
| 3 | 0.91 | 11.67 | 6.17 |
| 4 | 0.97 | 11.86 | 6.03 |
