# Supplementary material for: The regulation of oocyte maturation and ovulation in the closest sister group of vertebrates
Source: eLife. 2019 Oct 1;8:e49062. doi: 10.7554/eLife.49062 (PMC6786877; doi:10.7554/eLife.49062)
Supplement: Figure 5—source data 1. — Relative expression values of the CiMmp2/9/13 gene to CiUbac1 (RNA-seq and qRT-PCR data, Figure 5A and B). Percentages of ovulated follicles after incubating with CiVP and/or MMP-2/9 inhibitor II (Figure 5D and E). Relative expression values of the CiMmp genes to CiUbac1 (RNA-seq data, Figure 5—figure supplement 1). Raw values of in vitro collagenase activity of recombinant MMP-2/9/13 are also shown (Figure 5—figure supplement 3). [file elife-49062-fig5-data1.docx]

**Supplementary file 6.**

**Relative expression values of the *Ci-mmp2/9/13* gene to *Ci-ubac1* (qRT-PCR data).** Related to **Figure 5A.**

| **Independent experiment** | **Control** | **10 μM U0126** |
| --- | --- | --- |
| 1 | 1.28 | 0.40 |
| 2 | 0.56 | 0.28 |
| 3 | 1.16 | 0.19 |

**Relative expression values of the *Ci-mmp2/9/13* gene to *Ci-ubac1* (qRT-PCR data).** Related to **Figure 5B.**

| **Independent experiment** | **Control** | **5 μM Ci-VP** |
| --- | --- | --- |
| 1 | 0.97 | 7.54 |
| 2 | 2.41 | 4.68 |
| 3 | 0.39 | 1.57 |
| 4 | 1.30 | 4.99 |
| 5 | 0.44 | 2.48 |
| 6 | 0.48 | 2.94 |

**Percentages of ovulated follicles after incubating with a MMP inhibitor, MMP-2/9 inhibitor II.** Related to **Figure 5D.**

| **Independent experiment** | **Control** | **20 μM MMP-2/9 inhibitor II** |
| --- | --- | --- |
| 1 | 100.0 | 40.0 |
| 2 | 100.0 | 47.4 |
| 3 | 82.9 | 36.8 |

**Percentages of ovulated follicles after incubating with Ci-VP and/or MMP-2/9 inhibitor II.** Related to **Figure 5E.**

| **Independent experiment** | **Control** | **5 μM Ci-VP** | **5 μM Ci-VP **  **+20 μM MMP-2/9 inhibitor II** |
| --- | --- | --- | --- |
| 1 | 30.8 | 66.7 | 23.3 |
| 2 | 44.0 | 69.2 | 7.4 |
| 3 | 30.0 | 87.0 | 4.2 |
| 4 | 7.4 | 66.7 | 0 |
| 5 | 16.1 | 80.8 | 20.0 |

**Relative expression values of the *Ci-mmp* genes to *Ci-ubac1* (RNA-seq data).** Related to **Figure 5-figure supplement 1.**

| **Independent experiment** | **Control** | **10 μM U0126** |
| --- | --- | --- |
| ***Ci-DmMMP1 like a*** |  |  |
| 1 | 0.87 | 1.15 |
| 2 | 1.82 | 1.69 |
| 3 | 0.31 | 0.20 |
| ***Ci-DmMMP1 like b*** |  |  |
| 1 | 1.16 | 1.02 |
| 2 | 0.80 | 1.04 |
| 3 | 1.04 | 2.14 |
| ***Ci-DmMMP2 like*** |  |  |
| 1 | 0 | 1.10 |
| 2 | 0 | 1.70 |
| 3 | 0 | 0.19 |
| ***Ci-OrphanMMP*** |  |  |
| 1 | 0.83 | 5.03 |
| 2 | 0.50 | 3.00 |
| 3 | 1.66 | 5.72 |
| ***Ci-MMP-2/9/13*** |  |  |
| 1 | 0.45 | 0.07 |
| 2 | 1.01 | 0.20 |
| 3 | 1.54 | 0.26 |
| ***Ci-MMP-14/15/16/24*** |  |  |
| 1 | 0.60 | 0.89 |
| 2 | 0.85 | 0.60 |
| 3 | 1.55 | 1.60 |

**Relative values of *in vitro* collagenase activity of recombinant MMP-2/9/13.** Related to **Figure 5-figure supplement 3.**

| **Independent experiment** | **Control** | **rMMP-2/9/13** | **rMMP-2/9/13**  **+MMP-2/9 inh. II** |
| --- | --- | --- | --- |
| **Collagen type I** |  |  |  |
| 1 | 1.00 | 2.37 | 1.06 |
| 2 | 0.99 | 2.49 | 1.03 |
| 3 | 1.00 | 2.47 | 1.03 |
| 4 | 1.01 | 2.34 | 1.02 |
| **Collagen type IV** |  |  |  |
| 1 | 0.98 | 1.51 | 1.06 |
| 2 | 0.97 | 1.45 | 1.03 |
| 3 | 1.04 | 1.51 | 1.03 |
| 4 | 1.01 | 1.45 | 1.02 |
